# Explanation of the Colour Change in Alexandrites

**DOI:** 10.1038/s41598-020-62707-3

**Published:** 2020-04-09

**Authors:** Fei Xie, Yu Cao, Cindy Ranchon, Alan Hart, Robin Hansen, Jeffrey E. Post, Coralyn W. Whitney, Emma Dawson-Tarr, Alan J. Drew, David J. Dunstan

**Affiliations:** 1 School of Physics and Astronomy, Queen Mary University of London, London, E1 4NS UK; 2 College of Physical Science and Technology, Sichuan University, Chengdu, 610064 China; 3 Polytech Grenoble, Saint-Martin-d’Hères, 38400 France; 4 Natural History Museum, Cromwell Rd, London, SW7 5BD UK; 5 National Museum of Natural History, Smithsonian Institution, Washington, DC, 20560 USA; 6 Absolute Action Ltd, Horns Oak Farm, Meopham, DA13 0BX UK

**Keywords:** Physiology, Psychology, Solid Earth sciences, Materials science

## Abstract

Alexandrites are remarkable and rare gemstones. They display an extraordinary colour change according to the ambient lighting, from emerald green in daylight to ruby red in incandescent light from tungsten lamps or candles. While this colour change has been correctly attributed to chromium impurities and their absorption band in the yellow region of the visible light spectrum, no adequate explanation of the mechanism has been given. Here, the alexandrite effect is fully explained by considering the von Kries model of the human colour constancy mechanism. This implies that our colour constancy mechanism is real (objective) and primarily attuned to correct for the colour temperature of black-body illuminants.

## Introduction

Alexandrites are rare and highly-prized gemstones, which were first discovered around 1830 in Russia, and named after the future Czar, Alexander II, and later found in other countries such as Brazil and Sri Lanka^1^. They are prized for their dramatic colour change under different illumination: that is ruby-red under candlelight or incandescent lamplight, and emerald-green under natural daylight^1^. This is attributed to the Cr^3+^ impurities in the BeAl_2_O_4_ atomic structure which have strong optical absorption centred at the wavelength of yellow light. While the stones scatter blue, red and green light in proportions which vary with the illuminant^2–4^, this is not sufficient to explain the colour change in alexandrites as it is as true of any coloured object as it is of alexandrites. Here we give a complete explanation of the alexandrite effect. Taking into account the responses of the cone photoreceptors in the human eye, we show that the ratio of green to red stimuli under the different illuminants changes much more for an alexandrite than it does for normal coloured objects, which have broad absorption bands (inks, pigments, paints, fruit and flowers, etc). This overrides the mechanism by which the human visual system corrects for illuminance and helps understand the working of this mechanism for colour constancy. We expect these findings to have implications for theories of the colour perception of the human visual system in the somewhat disparate fields of traditional colour science^5^, the science of optical illusions^6^, and the study of individual perceptions, as in Impressionist paintings^7^ or the dress that went viral^8^.

## Results and Analysis

**Figure 1:**
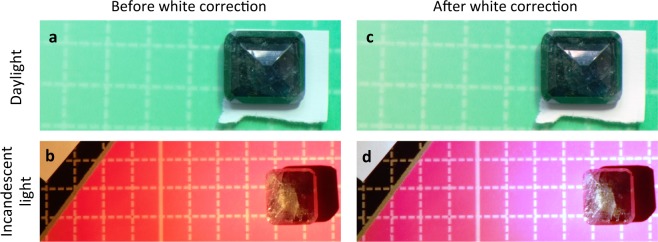
An alexandrite changing colour. The Russian alexandrite BM41178 from the collection of the Natural History Museum, London was photographed in (**a**) daylight and (**b**) incandescent light with the camera colour-correction feature switched off. The stone was placed on the matching colour on a printout of the CIE 1976 colour chart. A piece of white paper was included in the pictures, under the stone in (**a**) and nearby (top left) in (**b**). The corresponding images after colour balancing to make the paper white are shown in (**c**,**d**).

We studied some alexandrites from the collections of the Natural History Museum, London, and picked out two with a particularly pure and vivid alexandrite effect. One of these stones is shown in Fig. 1, under different illuminants, daylight and tungsten (incandescent) light. It was placed on a print of a CIE colour chart^9^ and moved to be on the matching colour as judged by eye (see Methods). With the camera’s colour-balancing software switched off, the effect on normal colours of the daylight is seen by the slightly bluish white paper (Fig. 1a) and of the incandescent light by the yellow-brown white paper (Fig. 1b). It is worth emphasising that to the observers under both illuminants the white paper appeared white, and the colours of the background colour chart appeared largely independent of the lighting. The brownish cast of the paper reflects only the brightness of the image, in that RGB yellow is close to (1, 0.9, 0) and just a 10% reduction in brightness to (0.9, 0.8, 0) gives brown. Colour balancing was optimised to create the corrected images (Fig. 1c,d, see the Methods section). The blue cast around the stone in Fig. 1d is discussed in the Supplementary §1.

A wide variety of physical phenomena from interference and diffraction to spectrally selective absorption can give rise to dramatic colour effects such as iridescence^10^. Explanations of the colour change in alexandrites have been given which correctly invoke the spectrally-selective Cr^3+^ absorption band in the yellow region of the spectrum^1–3^. Liu *et al*.^2^ took their explanation further, invoking also colour constancy. Experimentally, they said, the human visual system corrects for hue angle changes only up to 20° under different illuminations (hue angle is explained in the Supplementary §2). Calculating the change in hue angle for alexandrites and finding it to be greater than 20°, they attributed the absence of colour constancy in the alexandrite effect to that. However, the blue-to-brown hue angle change of the white paper in Fig. 1a,b is about 180°, yet colour constancy occurs. Recently a similar analysis of a weak alexandrite effect in purple flowers and low-quality alexandrite stones has been reported^11^.

It is necessary to consider more carefully what colour constancy does. Crucially, it works to the extent that to the human eye, the colours of the CIE chart under each lighting are almost unchanged. To get colour matches under the two illuminants, the stone has to be moved, and the colour matches are then recognisable to the eye and to the camera before and after correction. Objectively, to the camera as much as to the eye, the stone changes colour under different lighting. In contrast, pieces of blue, green, yellow or red paper do not have to be moved around the chart to keep the colour matches under different illuminations. Objectively, to the camera as much as to the eye, the pieces of paper do not change colour under different lighting.

Absorption spectra of two alexandrites R1 (BM41177) and R2 (BM41178) were recorded in a UV-VIS photospectrometer (Supplementary §3, Fig. S2). While these spectra were of low quality, because the stones are not optical flats, they confirmed the key features. Converted to transmission spectra, they show the Cr^3+^ absorption line centred on 572 nm with widths of approximately zero transmission of 90 and 120 nm, respectively, and also a strong absorption in the blue, cutting off all wavelengths less than about 480 nm, in agreement with the literature^1–4,11–13^. These data permit the prediction of what will be seen by the eye.

**Figure 2:**
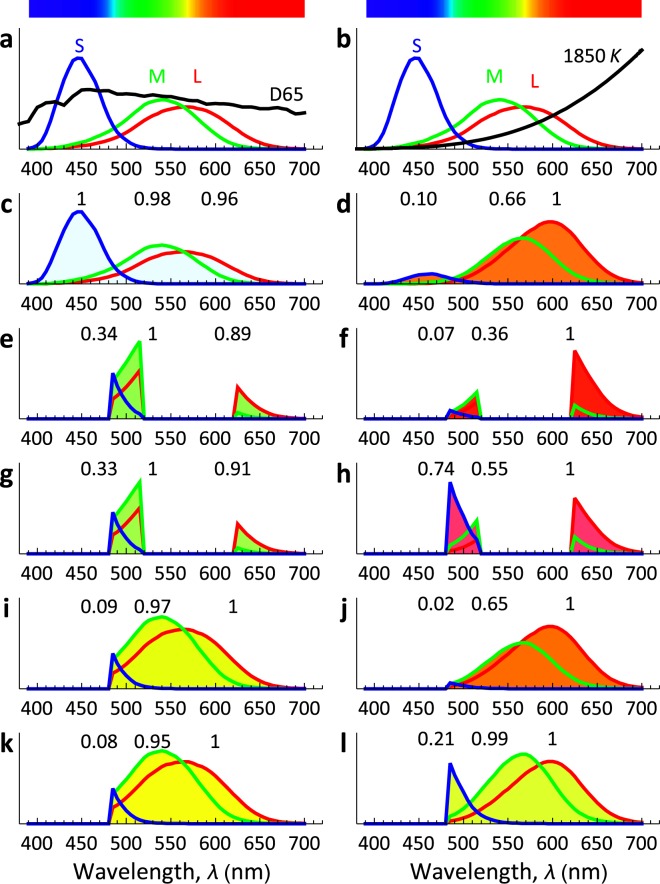
Analysis and explanation of the alexandrite effect. The CIE spectral response curves of the L, M and S cones are shown^14^, together with, (**a**) the daylight CIE D65 spectrum (colour temperature 6500 K)^14^ and (**b**) the candlelight black-body spectrum (1850K). In the panels below, for (**c**,**d)** a white object, for (**e**–**h**) the alexandrite R2, and, for **(i–l**) a yellow object, the filling colours are the colours calculated in LMS and converted to RGB, before colour constancy correction is applied in (**c,e,g**,**i**,**k**), and after (**d,f**,**h**,**j**,**l**). The LMS signals are marked above each component in (**c**–**l**). At the top, the visible spectrum calculated using monochromatic light and the same LMS-RGB correction is shown.

The human retina has three kinds of colour photoreceptors, or cones. The S cones detect short-wavelength light (blue), the M, medium wavelengths (green) and the L, long wavelengths (red). See Supplementary §4. In Fig. 2, the spectra of the responses of the cones, the illuminants and the transmittance of the stones (SI) are combined, in order to find out what is perceived. Figure 2 shows the spectral responses of the L, M and S cones^14^, with, Fig. 2a, the standard daylight spectrum D65^14^, and Fig. 2b, the candlelight spectrum (black body with a colour temperature of 1850K, which is approximately the standard illuminant A^14^). In Fig. 2c,d, the products of the illuminant spectra and the L, M and S spectra are plotted. The integrals of these curves correspond to the signals sent by the cones, and their values (normalised so the largest is 1) are marked. These LMS values are converted using an LMS-RGB conversion matrix (see Supplementary §5, Eq. S1) to the RGB colours used in the fill. These are approximately the colours that a white object would be perceived to have if we did not have colour constancy; they are also approximately the colours that an uncorrected camera records, as in Fig. 1a,b. In Fig. 2e,f, the wavelengths absorbed by the stones are removed from the illuminant spectra; what remains are the spectra of the light scattered by the stones. By daylight, the green (and some blue) dominates and the red is weaker; conversion to RGB gives the green used as fill in Fig. 2e. By candlelight the red dominates (Fig. 2f). Now we apply colour constancy, using a standard model of the mechanism, the von Kries correction^15^. See Supplementary §6 for details of our calculations and Supplementary §7 for a discussion of more advanced models^16,17^. The correction makes the colours of Fig. 2c,d white. Applied to the data of Fig. 2e,f, we get the data of Fig. 2g,h, where conversion to RGB again gives the fill colours.

The key point here is that the colour constancy correction hardly affects the daylight raw data (Fig. 2e), as D65 is close to flat across the visible spectrum, leaving the alexandrite green in Fig. 2g), and also does not affect the incandescent light raw data (Fig. 2f) sufficiently to change the colour of the alexandrite from red (Fig. 2h). In contrast, in Fig. 2i,j, the corresponding results are shown for a yellow object (absorbing short wavelengths to 480 nm and scattering all longer wavelengths through green, yellow and red). Here the uncorrected red in incandescent light is actually somewhat overcorrected beyond yellow to a greenish-yellow (due to uncertainties in the choice of RGB colour space, see Supplementary §5).

It is now clear what the physics of the alexandrite effect is. The daylight D65 spectrum is approximately flat, while 1850K blackbody falls off exponentially from the red to the blue (Fig. 2a). The M spectral response is so close to the L, only about 25 nm towards the blue, that this exponential weakens the M signal little (about one-third) relative to the L signal. In contrast, the S spectral response is so much further away, 120 nm, that it drops by nine-tenths relative to the L. These are the changes the colour constancy mechanism corrects when it has detected the illuminant. Under D65, the green light around 500 nm in the alexandrite spectrum dominates and the stone is green. Under the 1850K black-body illumination, after the alexandrite absorption has removed the light around 580 nm, the remaining green light around 500 nm and the remaining red light around 650 nm are so far apart that the green drops relative to the red enormously – much more than colour constancy will correct. Alexandrites do have many other interesting optical properties that may contribute to or detract from the effect, such as polarisation-dependent pleochroism^18^, but the wider separation of the remaining green and red in the spectrum after the stones have absorbed the yellow is clearly the fundamental explanation of the alexandrite effect.

**Figure 3:**
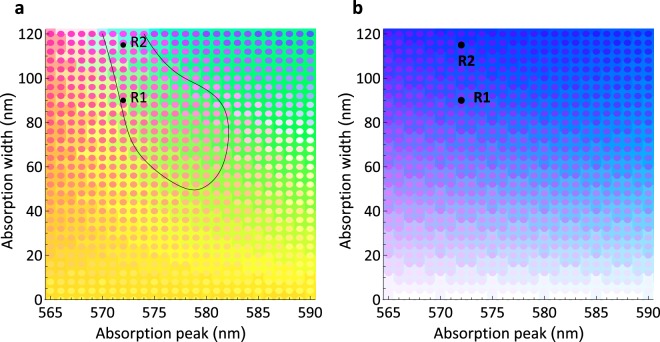
Mapping of the alexandrite effect. In (**a**), the colours calculated as for Fig. 2 for daylight (D65) and incandescent light (BB1850K) are plotted as a function of absorption peak position and peak width as background and overlaying spots, respectively. The small region where the full alexandrite effect occurs is outlined. The alexandrite stones R1 and R2 are marked on the map and fall within the outlined region. In (**b**) the map is calculated as for (**a**) but with the blue absorption band removed.

To directly see the alexandrite effect with different absorption peak positions and widths, a mapping based on Fig. 2 of calculated RGB colours is shown in Fig. 3a. Both stones R1 and R2 are positioned where clear red/green colour contrast occurs (for more details see Supplementary §8 Fig. S4). The importance of the blue absorption is shown by the map in Fig. 3b, where the blue cut-off has been removed from the absorption spectrum. Now, the alexandrite effect is largely eliminated. Only the weak blue/purple and blue/green colour transitions characteristic of the chrysoberyls^2^ – and some purple flowers^11^ – remain.

The importance of the yellow absorption for the alexandrite effect is confirmed by our analysis. The very narrow limits on both its position and its strength (i.e. width) if a true red – green alexandrite effect is to be observed are striking. Taking these requirements together with the previously unreported requirement for a blue absorption, it is not surprising that natural alexandrites are so rare, and so prized. Indeed, given this analysis, for large stones or stones with too high a chromium doping in which the true red-green alexandrite effect is weakened by too little red and green transmission compared with the blue, we can conclude that by reducing the yellow absorption-band width, one would be able to increase the alexandrite effect. This could be achieved by cutting the stones thinner (Supplementary §10).

## Discussion

These results have impact for traditional colour science, in which colour constancy and the related ability to discount the illumination, are sometimes considered to be approximate at best, or even fundamentally non-existent^19^. Yet the alexandrite effect may be described as an objective colour change just as the appearance of yellow under different illuminants may be described as objective constancy. Both can be verified by colour matching by eye and by RBG values (as in the raw and corrected photographs in Fig. 1), consistent with the colorimetry matching that underpins colour science. This questions why a yellow object does not show a colour change but alexandrites do. For people with normal colour vision, yellow is a different *quale* from red and green^20^, i.e. “yellow” is a basic colour term describing a unique rather than a binary hue such as greenish-blue or bluish-green^21^. There is no reddish-green or greenish-red. Physiologically, yellow is perceived only when the amount of the L and M stimuli to the eye are very close to equal (and greater than the S stimulus)^22^. Yet the eye-brain system is so sophisticated that it normally sees a yellow object as yellow even when incandescent light or candlelight swing the red and green stimuli far away from their ratio under daylight. The precision of this correction can be judged from Fig. 2. The ML ratio of 0.66 in Fig. 2d is corrected to white, and would be corrected to yellow if the S signal were weaker, while the (not dissimilar) ML ratio of 0.59 in Fig. 2f is corrected to ruby-red. From Fig. 2c,l we see that the correction is made to an accuracy of 1% or better. It is plausible that the input data is the ratio of S to L + M, for a rough estimate of that will gives a very good estimate of the ML ratio for black-body illumination^23^. So we conclude that the human visual system does have – must have – a very efficient colour constancy mechanism, *provided* that it is black-body colour temperatures that are to be corrected, and that the mechanism acts *before* the L and M signals are compared.

This also links to the question: whether colour constancy (to the extent it exists) is innate or learned. One might expect that millions of years of evolution of dichromatic mammals with only two kinds of cones, S and ML) under essentially black-body illumination would lead to innate colour correction based on the ratio of the S to ML. Following this reasoning to consider trichromacy, we test how the required changes in the sensitivities of the M and L cones, for black-body white-light illuminants, might be related to the S signal. Calculations (as for Fig. 2, and detailed in Supplementary §7) give the curves of the three signals S, M and L as a function of colour temperature from 1850K to 12000 K. Rather surprisingly, the corrections for M and L turn out to be linearly related to the logarithm of the change in S (Supplementary Fig. S3). Noting that most human sensations scale logarithmically with the stimulus (cf. the decibel scale for sound), we speculate that this adaptation of the M and L sensitivities, linear with the S sensation and arising only relatively recently with trichomacy, would be implemented at a higher level of the processing of the signals than the raw S v. ML correction.

Another important correction that our visual system does, that silicon-based colour correction does not normally do, is to correct the colour in a shadow to the colour of the unshadowed parts of the same object. The Impressionists were the first artists to observe that shadows are in fact different colours (by overriding their own colour constancy mechanism) and to paint shadows accordingly^7^. Initially, in the 1870s, this shocked the critics and the public; now we view the shadows in these paintings without any sense of shock, implying that there is a learned component to colour constancy. One aspect that could be learned rather than innate is the ability when viewing a painting, a photo or a screen to let colour constancy operate within the picture independently of ambient lighting and the surrounding colours. The extent to which different people learn to do this could account for the different responses to the picture of the dress that went viral^8^. Specifically, the white parts of the dress are a blue very similar to the blue of the white paper in Fig. 1a. Some peoples’ colour constancy (or ambient lighting) may correct this to white if they are used to such images, and the same correction then takes the other parts to gold. Others, with different visual histories (or ambient lighting), clearly seeing the blue, will then rescale the other parts of the dress to black. However, following Foster^16^, we suppose that all these corrections come after the initial von Kries correction for the illuminant colour temperature.

In summary, the colour balancing and correction of the photographs of alexandrites and the calculations of the colour change based on colorimetry and colour space theories indicate that the precision of the colour constancy mechanism in the human eye-brain system and its overriding by the alexandrites play the key role for the alexandrite effect. These results provide a broader insight into colour constancy and colour perception in the human eye-brain system.

## Methods

The alexandrite stones (BM41177 and BM41178, both from Russia) were from the collection of the Natural History Museum, London, UK. They were observed and photographed under natural daylight (north light, in the shadow of a large building on a summer day with sun and scattered clouds) and in a darkroom with only incandescent light for illumination. Candlelight and a tungsten lamp were both used with very similar results; the images in Fig. 1b,d were made with the tungsten lamp. The stones were placed on a printout of a CIE 1976 UCS colour chart^9^ as a background, and a piece of white paper was placed in the picture. The position was selected by five judges (CR, AH, EDT, AJD and DJD for one session and AH, RH, EDT, AJD and DJD for another session). Each judge moved the stone as they thought best to improve the colour match to the chart; this process converged each time on a position at which no-one proposed a further move, i.e. a position to which all judges consented. A digital camera (Nikon D5100, 16 MP CMOS detector) was used with all colour-balance and selective-colour software switched off, and flash and autofocus also off. Images were exported from the camera as NEF files (raw 14-bit data from the image sensor) and converted to JPGs by opening them in Microsoft Photos and saving a JPG copy.

These files were then imported into *Mathematica*^©^ notebooks using image = Import[filename], and converted to two-dimensional tables of triplet {R, G, B} values using data = ImageData[image]. Parts of the images could be selected using Part[data,{indices}].

Colour balancing was applied to create the corrected images (Fig. 1c,d). For Fig. 1, we used a simple approximate technique for RGB image files. These photos were corrected by dividing all pixel (R, G, B) values by the (R, G, B) values of the white paper. Then the image was re-created using image = Image[data]. This makes the paper RGB-white, (1, 1, 1), in the colour space of the camera and of any RGB or CMYK display unit or printer (see Supplementary §1). The procedure is not very accurate for other colours because the response curves of the R, G and B photodetectors in the camera are not known nor used, and nor are the spectra of the R, G and B light sources in a computer monitor or the corresponding spectra of the inks in a colour printer. In particular, the blue cast of the red CIE chart in Fig. 1d around the stone, where the light was brightest, appears to arise from a sub-linearity of the photodetectors at high light levels (see Supplementary §1).

## Supplementary information

Supplementary Information.
