# Supplementary material for: Explanation of the Colour Change in Alexandrites
Source: Sci Rep. 2020 Apr 9;10:6130. doi: 10.1038/s41598-020-62707-3 (PMC7145866; doi:10.1038/s41598-020-62707-3)
Supplement: Supplementary file 1 — Supplementary Information. [file 41598_2020_62707_MOESM1_ESM.pdf]

Supplementary Information

Explanation of the Colour Change in Alexandrites

Fei Xie, Yu Cao, Cindy Ranchon, Alan Hart, Robin Hansen, Jeffrey E. Post, Coralyn W. Whitney, Emma Dawson-Tarr, Alan J. Drew, and David J. Dunstan

Section 1. Colour balancing in Fig.1. None of our photos showed evidence of over-exposure. The correction factors were division by the white paper RGB values, daylight (0.80, 0.96, 0.96) and incandescent (0.84, 0.58, 0.31), followed by normalisation to avoid values greater than 1. However, there is a blue cast around the stone in the corrected Fig.1d. Fig.S1 shows plots of the RGB components across the uncorrected photographs. The uncorrected white paper and the corrected stone are shown in each plot at the relevant positions. The stones are lightened by multiplying all RGB values by 3 to make the green or red colour of the darker parts more obvious. In Fig.S1a, there is no evidence of saturation or non-linearity of the photodetectors. In contrast, in Fig.S1b for incandescent light, it is clear that the red photodetector is saturating, with a very sublinear response above 0.8. However, there is no green or blue in the stone.

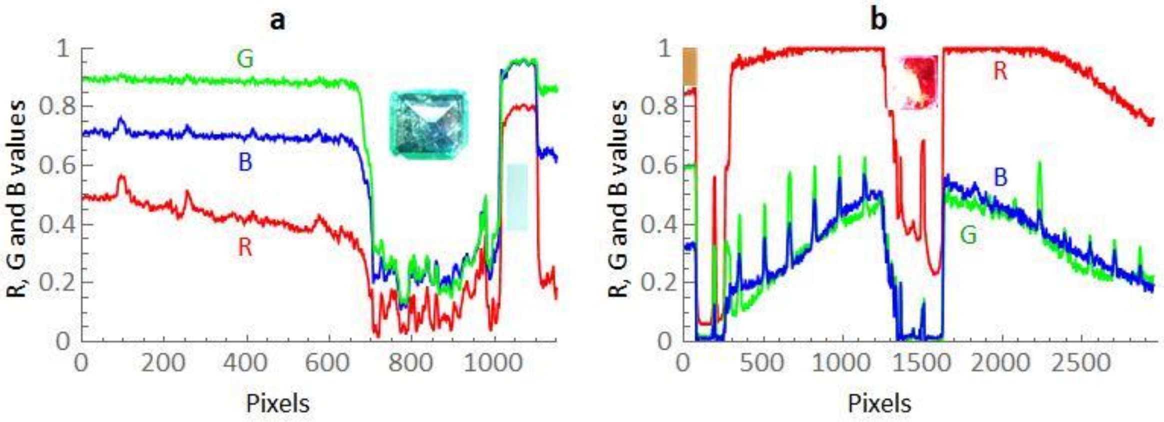

Fig. S1. RGB values from Fig.1. A line of pixels from left to right from the uncorrected images Fig.1a (a) and Fig.1b (b) are plotted. The lightened corrected images of the stones are shown, at 3 × RGB, centred on pixel 900 (a) and 1500 (b). Uncorrected images of the white paper are centred on pixel 1100 (a) and 50 (b).

Section 2. Hue angle and colour theory: Newton proposed five, and later seven primary colours; pure colours which he identified in the rainbow.[S1] Yellow was one of these. With paints, pigments and printers, blue (or cyan), yellow and red (the CMY subtractive colour space) are primary colours from which others, e.g. green can be made by mixing yellow and blue. However, looking at the light coming from a yellow pigment such as chrome yellow, it is not for the most part yellow light; instead the pigment works by absorbing (subtracting) the blue component of white light and scattering the rest of the spectrum (predominantly green and red light). In natural white light, there is a little yellow light, in the remarkably narrow spectral range around 575 – 585 nm, but generally a pure yellow object looks pure yellow not because it scatters yellow light (though it does) but because it scatters green and red light equally. Indeed, a pigment that absorbed all other light and scattered only the yellow light in the 575 – 585 nm range would not look yellow, but rather dark brown (brown is dark yellow). For emissive displays, in contrast to pigments, colours are added. Generating yellow for a display is done by generating green and red; adding blue as well gives white. This is the basis of the RGB additive colour space.[S3]

The hue, saturation, lightness (HSL) model is a representation of colour spaces in which colours are arranged in a circle opposite their complementaries and a colour can be described by a single number, the angle.[S3, S4] Typically, if blue is centred at 0º, yellow is centred at 180º, with green and red centred near 90º and 270º, cyan is between blue and green, orange is between yellow and red, and purple is between red and blue.

Section 3. Absorption Spectra

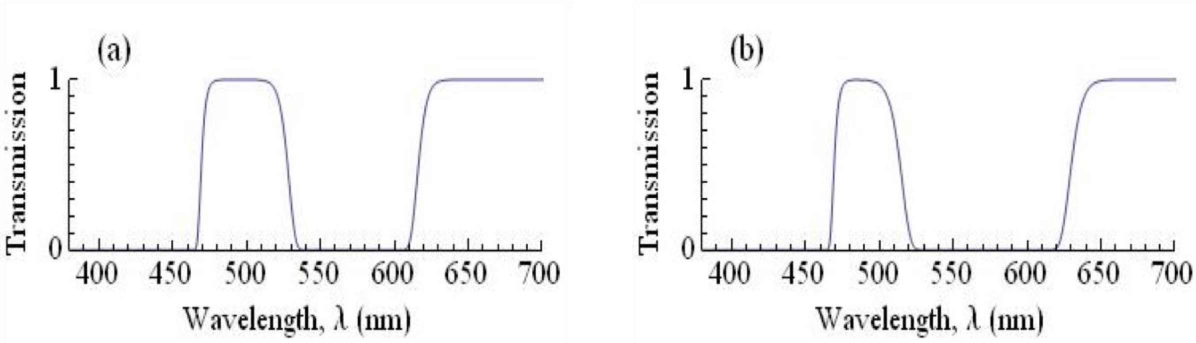

Fig. S2. The transmission spectra of alexandrite stones from the Natural History Museum, for (a) stone R1 (BM 41177) and (b) stone R2 (BM 41178). These spectra are calculated from fits to the noisy experimental absorption spectra.

Section 4. LMS colour space: While the human eye has red, green and blue receptors (cones), they are not well described as a simple RGB sensor system (as in a camera). Instead, the cones are better described as long, medium and short wavelength receptors (LMS colour space). Their spectral sensitivities $L(\lambda)$, $M(\lambda)$ and $S(\lambda)$ are known.[14] They are plotted in Fig.2 (a), normalized to equal areas so that white will be (L, M, S) = (1, 1, 1). The response curves of the red (L, long wavelength) and green (M, medium wavelength) cones are surprisingly close together. Only near equality (to a few %) of the L and M responses gives yellow, while even small excesses of the M or L responses give green or red.

Section 5. LMS-RGB conversion matrix: Conversion of colours calculated in the LMS colour space to an RGB colour space requires an RGB-LMS conversion matrix. There are many definitions of the RGB colour space and consequently numerous conversion matrices exist in the literature. The first well-defined RGB colour space used primaries based on three sharp lines of monochromatic light from discharge lamps.[S2] Solid-state laser projectors can have other choices of sharp wavelengths, but on the other hand visual display units and colour printers necessarily use broad wavelength ranges for the primaries. We used the three wavelengths $\lambda_B = 450$ nm, $\lambda_G = 515$ nm and $\lambda_R = 620$ nm. First the RGB-LMS matrix is found. Taking each of the three monochromatic RGB sources in turn, the nine terms $L(\lambda_i)$, $M(\lambda_i)$, and $S(\lambda_i)$, i = R, G, B, are written as a 3×3 matrix. The inverse of this matrix is the RGB-LMS matrix,

$$\begin{bmatrix} R \\ G \\ B \end{bmatrix} = \begin{bmatrix} 2.71667 & -1.77027 & 0.05360 \\ -0.34944 & 1.53261 & -0.18317 \\ 0.00956 & -0.04194 & 1.03238 \end{bmatrix} \begin{bmatrix} L \\ M \\ S \end{bmatrix} \qquad \text{(S1)}$$

Section 6. Von Kries correction: For Fig. 2, we used the von Kries method.[16,18] Daylight may be approximated by a black-body curve for 6000K (BB6000K), but is more accurately represented by various standard CIE illuminants, of which we used D65 (average daylight) with a colour temperature of 6500 K.[15] Incandescent light (ordinary tungsten filament bulbs)

corresponds quite accurately to black-body spectra, and the CIE standard illuminant A for tungsten bulbs has a colour temperature of 2850K (BB2850K). There is no standard for candlelight but black-body 1850K (BB1850K) is a reasonable approximation.

The L, M and S responses are calculated by multiplying the illuminant spectra by the response curves and integrating to get I_L, I_M and.I_S. The reciprocals of these numbers are the diagonal elements of the correction matrix; the off-diagonal elements of which are zero. Then an LMS colour is corrected for the illuminant by multiplying the (L, M, S) vector by the correction matrix.

Our use of monochromatic RGB sources to generate the LMS-RGB conversion matrix is probably responsible for the slight overcorrection for candlelight (the yellow is a bit too green in Fig. 2l). That is due to the width of the real display unit or print RGB spectra, and it will be what creates the difficulty in getting a pure red for the alexandrite in Fig.2h.

Section 7. Other corrections: Much more complicated and accurate models of colour constancy have been developed, most of which however have the von Kries hypothesis as their basis.[17,23] They generally address two points that von Kries leaves unspecified. First, how the human visual system might establish what the illuminance is. Examples are the "bright is white" assumption, and the space-average chromaticity or grey-world assumptions.[16] Second, context and interactions between spatially-separated parts of the visual field. Also mechanisms have been proposed, such as that the cone responses adapt to the stimulus – but Foster notes that this can have the difficulty that it would predict perfect colour constancy for all changes in the illuminant.[16]

In our work, we know the illuminants and we include white paper in the photographs. We make only local, not global measurements. Accordingly, the von Kries rule applied as we do should be sufficient.

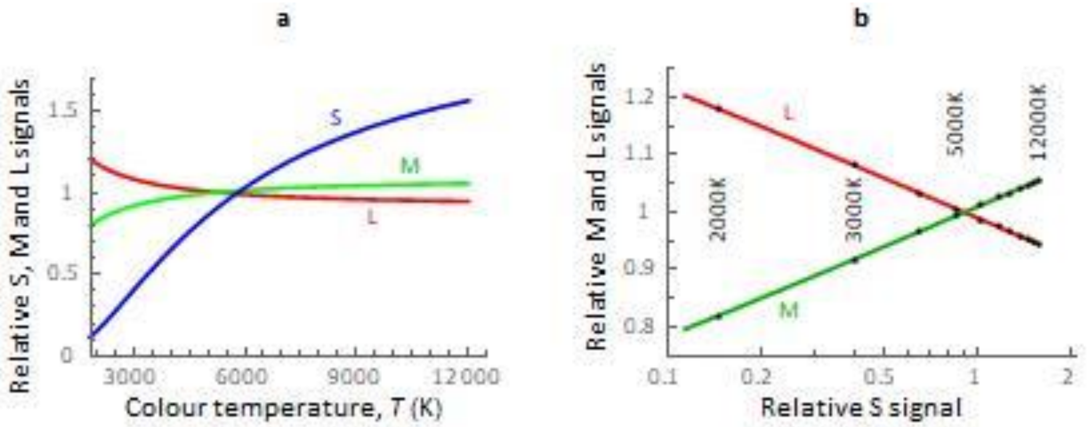

Fig. S3. Cone responses and corrections for black-body colour constancy. In (a), the cone signals S(T), M(T) and L(T) are normalised to (1,1,1) at 5500K. In (b) M(T) and L(T) are plotted against $\log_{10}$S(T); the black points mark the temperatures 2000K – 12000K. The data fit accurately straight lines, 1 ± 0.23 $\log_{10}$S(T), indicating that the required changes to the M and L sensitivities are proportional to the perceived blue content of the scene or the light.

It is noteworthy – with implications for mechanisms of colour constancy - that the Alexandrite effect is very robust and independent of the rest of the field of view, with black-body or near-black-body illuminants – daylight, tungsten lamps and candles. In contrast, it is difficult or impossible to obtain with other white light illuminants (see Section 9 below), under which stones may fail to change colour or change colour unpredictably. Thus our colour-

constancy mechanisms do not work reliably for non-black-body illuminants. We speculate that this implies a robust colour-constancy mechanism that will have evolved specifically for black-body illumination, and which will underly the other corrections mentioned above. Given the relatively recent evolution of trichromacy, it is plausible that this black-body von Kries mechanism is functionally equivalent to changing the gain (sensitivity) of the L and M cones in proportion to the ratio of the S signal to the L and M signals. Calculating the S, M and L responses as in Fig.2, but for black-body white light as a function of colour temperature we get the curves of Fig.S3a. Replotted as the parametric plot of Fig.S3b, the M and L responses turn out to vary linearly with the logarithm of the S response, or linearly with the sensation. Many human sensations are logarithmic with the stimulus (hence the decibel for sound). M and L cone responses adapting in this way to the blue sensation would indeed correct perfectly for black-body illuminants but not others.

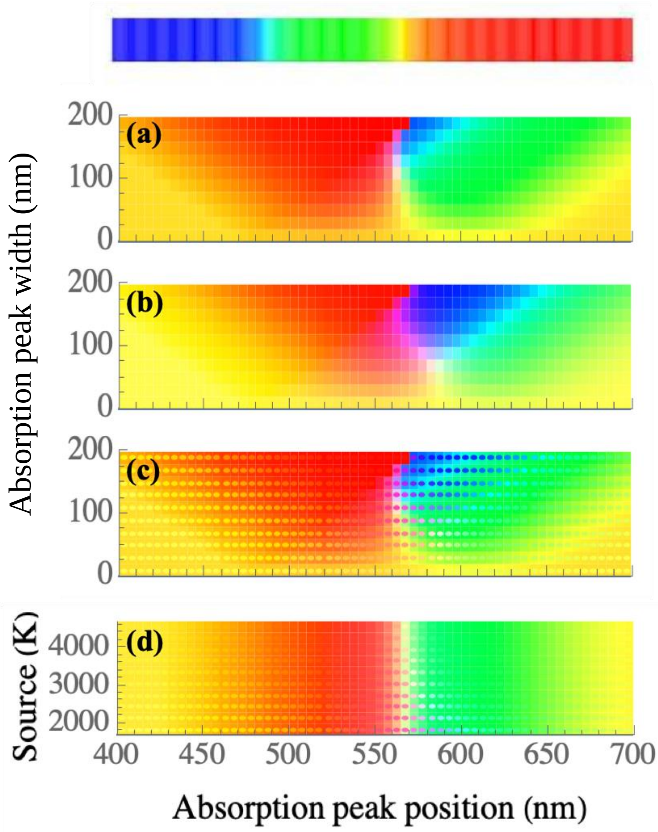

Fig. S4. Mapping the Alexandrite Effect. In (a), the colours calculated as for Fig.2 for daylight (D65) are plotted as a function of absorption peak position and peak width. The corresponding colours by candlelight (BB1850K) are plotted in (b). In (c), the candlelight colours of (b) are shown in small spots overlaying the background of daylight colours of (a). Finally, the effect of the candlelight colour temperature is shown in (d), in which the vertical axis is the colour temperature for the candlelight spots while the background is for the D65 daylight. Here, contrast disappears at about 4000K. The inaccuracy of the colour rendering in this Figure can be estimated by comparing the calculated spectrum shown above (a) with any true rainbow.

Section 8. Mapping: Using the same method as for Fig. 2, the perceived colours have been calculated as a function of the peak position and widths of the yellow absorption band, with the results shown in Fig. S4. Figure S4a is for a stone illuminated by daylight, and it is clear

that if the absorption peak is narrow or weak the stone is yellow, as expected for anything with a dominant blue absorption. When the absorption peak is wide, strong, and in the green, the stone is red, while if it is in the red, the stone is green (see the rainbow spectrum above Fig. S4a). The transition between these is remarkably sharp. If the absorption is too broad, the red and green are both removed sufficiently to leave the stone blue. The same analysis describes Fig. S4b, which is the same map but calculated for candlelight. The only significant difference from Fig. S4a is that the transition through yellow is shifted to longer wavelengths. The blue portion at large peak widths is also larger. Although the alexandrite effect is seen by comparison of the two maps shown in Figs. S4a and b, a more much intuitive way to observe it is to superimpose the two maps on top of one another. This is done in Fig. S4c, where the candlelight map of Fig. S4b is superimposed as small spots on the daylight map of Fig. S4a. The most relevant part of this plot is shown at higher resolution in Fig. 3a in the main text. It is apparent that the true alexandrite effect (red spots on green background) is observed only for a very limited range of peak positions and widths, as marked on the Figure. A larger region of green – blue contrast is also observed, and there are regions of red – yellow and purplish contrast.

Section 9. Non-Incandescent Lamps:

This analysis also explains why alexandrites are in practice difficult to light. Lighting in museums and galleries has changed in recent years from largely daylight and incandescent light, to much more efficient and reliable artificial sources – fluorescent tubes and more recently LEDs. Absolute Action Ltd. used metal-halide arc lamps with optical fibre to light various coloured gemstones with excellent results, using 4000°C lamps for the Hope diamond (blue) and the Dresden diamond (green), and 3000°C lamps for the Hooker starburst diamonds (yellow), the Steinmetz diamond (pink) and the Moussaieff diamond (red), among others. However, with these lamps and colour temperature correction filters, it was difficult to obtain the alexandrite colour transition to red. Much of the light from these lamps is concentrated into a few sharp peaks as in Fig. S5. A wider range of colour temperatures is achieved using commercial filters. Generally these, too, have quite peaky spectra (Fig. S5), so while they achieve their nominal colour temperature change accurately on black-body light, and more-or-less well on other white light such as metal-halide, there is no guarantee that the final ratios of the light intensity in the two rather narrow bands of relevance in Fig. 2c are as required to replicate either daylight or candlelight. Getting the illumination correct to show the alexandrite effect is therefore challenging. A key issue is the 475nm peak in the lamp spectrum (Fig. S5), and whether a particular stone attenuates it a little or a lot.

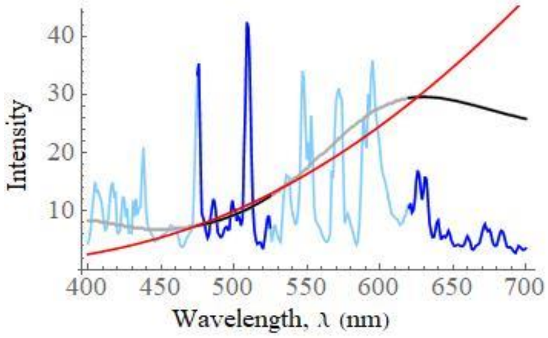

Fig. S5. A metal-halide arc-lamp spectrum is shown in blue; the two bands of relevance around 500 nm and 650 nm are in dark blue. The absorption spectrum of a typical commercial filter for changing colour temperature is shown in grey, black in the regions of relevance. This filter is designed to convert 5500K white light to

2700K. The red curve is the theoretical spectrum for this colour temperature change. With this lamp and the actual spectrum of the filter, it converts the colour temperature defined by just the 500 nm and 650 nm bands from about 20000K to about 6000K.

Section 10. Thinning: The chromium absorption in Fig.S1 is saturated, i.e. strong enough that the transmission is effectively zero around the peak absorption. In this case, decreasing the chromium content, or equivalently thinning the stone, weakens the absorption and has the effect of narrowing the band of zero transmission. This could improve the performance of stones showing a purplish – green-blue alexandrite effect.

Supplementary References (other references are in the list in the main text).

S1. Newton, I. A letter of Mr. Isaac Newton, Professor of the Mathematicks in the University of Cambridge; containing his new theory about light and colors: sent by the author to the publisher from Cambridge, Febr. 6. 1671/72; in order to be communicated to the R. Society. *Philos. Trans. Roy. Soc.* (1671). doi:https://doi.org/10.1098/rstl.1671.0072

S2. Wright, W. D. A re-determination of the trichromatic coefficients of the spectral colours. *Trans. Opt. Soc.* **30**, 141–164 (1929).

S3. Schanda, J. *Colorimetry: Understanding the CIE System*. (Wiley Interscience, 2007).

S4. Fairchild, M. D. *Color Appearence Models*. (Wiley, 2005).
